# 2795. Cefepime Versus Meropenem for Infections Due to HECCcK-YeS-MAm Organisms with Cefepime MICs of 4-8 mCg/mL (S-DD)

**DOI:** 10.1093/ofid/ofad500.2406

**Published:** 2023-11-27

**Authors:** Andrew Scott Purdy, Zach Howe, Armisha Desai

**Affiliations:** Indiana University Health, Indianapolis, Indiana; Indiana University Health Riley Hospital for Children, Indianapolis, Indiana; Indiana University Health Adult Academic Health Center, Indianapolis, IN

## Abstract

**Background:**

Recent guidance from IDSA recommends meropenem for treatment of organisms at moderate-high risk for inducible chromosomal AmpC β-lactamase hyperproduction (AmpC organisms) with a cefepime minimum inhibitory concentration (MIC) of 4-8 mCg/mL (susceptible dose-dependent or S-DD).1 This runs contrary to pharmacokinetic/pharmacodynamic data and Clinical and Laboratory Standards Institute (CLSI) M100 guidance, which considers organisms ≥ 16 mCg/mL resistant.2 The purpose of this study is to evaluate the clinical efficacy of cefepime versus meropenem for the treatment of infections caused by AmpC organisms with a S-DD cefepime MIC.

**Methods:**

This was a retrospective study evaluating patients treated within Indiana University Health from 2015-2022. All cefepime S-DD AmpC isolates were included. Exclusion criteria were < 48 hours of treatment, multiple days of therapy with another active agent, inadequate dosing, extended spectrum β-lactamase (ESBL) co-production, and carbapenem resistance. The primary endpoint of this study was a composite of in-hospital all-cause mortality and antibiotic escalation due to clinical worsening. Secondary endpoints include positive repeat blood cultures within 7 days of treatment initiation, and ESBL or carbapenem resistant organism isolation during or following treatment.

**Results:**

355 isolates were screened. 71 patients were included in the final analysis, 43 in the cefepime group and 28 in the meropenem group. Baseline characteristics were not significantly different. Enterobacter cloacae made up 79% of cases. The primary endpoint occurred in 13/43 (30%) of patients on cefepime and 7/28 (25%) of patients on meropenem (P=0.63). Treatment escalation and escalation due to clinical worsening was higher in the cefepime group with 7 (16%) and 6 (14%) incidents respectively compared to none in the meropenem group (P=0.03; P=0.04). No other endpoints, including all-cause mortality, were significantly different between groups.

**Conclusion:**

Cefepime remains a viable treatment option for ESBL-negative, AmpC organisms with MICs in the S-DD range.

**Disclosures:**

**All Authors**: No reported disclosures

